# Follow-up of patients with chronic conditions within primary care practices during COVID-19: Results from 7 Central and Eastern-European countries from the cross-sectional PRICOV-19 study

**DOI:** 10.1080/13814788.2024.2391468

**Published:** 2024-08-29

**Authors:** Giulia Delvento, Christian Schindler, Cristina Rotaru, Ala Curteanu, Ghenadie Curochicin, Helen Prytherch, Victoria Tkachenko, Bohumil Seifert, Peter Torzsa, Radost Asenova, Carmen Busneag, Adam Windak, Sara Willems, Esther Van Poel, Claire Collins

**Affiliations:** a Swiss Tropical and Public Health Institute, Allschwil, Switzerland; b University of Basel, Basel, Switzerland; c Healthy Life Project, Reducing the Burden of Non-Communicable Diseases in Moldova, Swiss Agency for Development and Cooperation (SDC), Chișinău, Moldova; d Mother and Child Institute, Chișinău, Moldova; e Department of Family Medicine, Nicolae Testemitanu, Chișinău, Moldova; f Department of Family Medicine, Shupyk National Medical Academy of Postgraduate Education, Kiev, Ukraine; g Institute of General Practice, First Faculty of Medicine, Charles University, Prague, Czech Republic; h Department of Family Medicine, Semmelweis University, Budapest, Hungary; i Department of General Medicine, Plovdiv University, Plovdiv, Bulgaria; j Department of Kinetic Therapy and Special Motricity, Spiru Haret University, Bucharest, Romania; k Department of Family Medicine, Jagiellonian University Medical College, Krakow, Poland; l Department of Public Health and Primary Care, Ghent University, Ghent, Belgium; m Quality and Safety, Ghent University, Ghent, Belgium; n Irish College of General Practitioners, Dublin, Ireland

**Keywords:** Patient follow-up, outreach, quality of care, COVID-19, multi-country

## Abstract

**Background:**

The COVID-19 pandemic posed severe challenges to delivery of services at Primary Care level and for achieving follow-up of patients with chronic diseases.

**Objectives:**

We analysed data from the PRICOV-19 study to explore determinants of active follow-up for chronic disease patients in seven Central and Eastern European (CEE) countries during the pandemic.

**Methods:**

Pricov-19 was a cross-sectional study conducted within PC (Primary Care) practices in 37 European countries. We analysed data from 7 CEE countries (Bulgaria, Czech Republic, Hungary, Poland, Moldova, Romania, Ukraine) collected between November 2020 and December 2021. Practices were recruited through random or convenience sampling and participation of practices was voluntary. We performed descriptive statistics to identify the level of follow-up of chronic disease and what health system and practice-specific factors were associated with better follow-up. We used logistic regression and meta-analysis techniques to explore associations and heterogeneity between countries.

**Results:**

67.8% out of 978 practices reported actively following up chronic patients. Positive associations were found between active follow-up and such as having more GPs (aOR = 1.18, p-value = 0.005), an above-average chronic patient population (aOR = 3.13, p-value = 0.006), adequate government support (aOR = 2.35, p-value = 0.001), and GPs having time for guideline reading (aOR = 0.008, p-value = 1.71).

**Conclusions:**

Patient follow-up, was influenced by different health system and practice-specific factors. The implications suggest the need for government support to enhance PC practice organisation during crises and solutions to decrease GP workload and provide tailored care for patients with chronic disease.

## KEY MESSAGES

In 7 Central and Eastern European countries, 68% of PC practices effectively followed-up patients with chronic conditions during the pandemic.

Key determinants for successful follow-up included government support, GP time availability, and staffing levels of GPs.

Video consultations and payment mechanisms did not show significant associations with optimal patient follow-up.

## Introduction

The COVID-19 pandemic disrupted healthcare delivery worldwide, including within the most well-resourced and advanced health systems [1, 2]. In the early phases of the pandemic, there was a lack of attention and investment into primary healthcare (PHC), due to funds diverted towards supporting overburdened hospitals struggling to remain functional during peak increases in COVID-19 cases [3]. At PHC level, reductions in access to care for patients with chronic disease during COVID-19 have been observed across different studies, potentially leading to treatment delays [4–9]. Routine visits at the primary care level were postponed thus contributing to treatment delays or missed diagnoses [10, 11]. Other factors may also be linked to poor patient follow-up as evidenced in other studies, such as the number of staff in the primary care (PC) practice and the type of payment system in place for doctor’s remuneration [12]. Both of these factors can also impact the time availability of the GP for actively following up chronic disease patients due to an increase in workload which has also been demonstrated to be a potential determinant of poor follow up [13]. Governmental support in times of crisis has also been shown to be crucial to ensure adaptation of PC practices to emerging needs brought by the pandemic and the rapid adoption of new telemedicine technologies [14].

In this paper, we describe determinants of active follow-up at PC practices for patients with chronic disease within seven Central and Eastern European (CEE) countries, namely Bulgaria, Czech Republic, Hungary, Republic of Moldova, Poland, Romania, and Ukraine. Active follow-up was defined as the GP practice proactively contacting patients in need of chronic care during the COVID-19 pandemic.

These CEE countries have experienced important reforms of the health care sector and in primary health in the past years and have made important progress towards expanding universal health coverage, by reducing the incidence of catastrophic health spending and progressively strengthening their health systems at a primary care level. Despite the progress, issues remain at different levels, for instance, with the retention of the health workforce and the increasing need for primary care services for increasing numbers of older age adults living with chronic conditions. In many Eastern European countries there is also an added high burden of preventable diseases, and a high prevalence of older adults living with 2 or more comorbidities (for more details on the characteristics of single countries, see Table 1). CEE countries have a high burden of chronic disease compared to other European countries due to different age distributions and socio-economic factors in their population, and a higher prevalence for cardiovascular diseases (CVDs) linked to lifestyle factors such as smoking, dietary factors and low levels of physical activity [15–20].

**Table 1.** Health system characteristics and indicators of the 7 countries involved in the study.

Country	Population, total	Current health expenditure (CHE) as % gross domestic product (GDP)*	Out-of-pocket (OOPS) as % of current health expenditure (CHE)*	Primary health care (PHC) expenditure as % current health expenditure (CHE)*	Physicians per 1000 people**	Type of health system	Prevalence of population over 65 with 2 or more chronic conditions (a)
Bulgaria	6.975.761	7	39	NA	2.6	Social Health Insurance	38%
Czech Republic	10.671.870	8	14	32	4.1	National Health Insurance (Bismark model)	41%
Hungary	9.771.141	6	28	41	3.3**	National Health Insurance (Bismark model)	60%
Poland	37.965.475	6	20	43	3.5	Mixed (Fragmented HS)	56%
Republic of Moldova	2.664.974	6	36	46	4.1	Social Health Insurance	NA
Romania	19.371.648	6	19	36	3.2***	National Health Insurance (Bismark model)	32%
Ukraine	44.386.203	7	51	NA	4.17****	National Health Service	NA

*WHO Global Health Data Observatory Indicators (who.int).

(a) OECD/European Union (2022), Health at a Glance: Europe 2022: State of Health in the EU Cycle, OECD Publishing, Paris, https://doi.org/10.1787/507433b0-en, data from 2021.

Additionally, CEE countries are also affected by shortages of healthcare workers due to ageing of staff and a preference of young staff to emigrate in search of better working conditions and work-life balance [21].

We explored if determinants such as characteristics of the PC practice, individual general practitioner (GP), the presence of specific interventions implemented during COVID-19 and macro-health system factors were associated with active follow-up of chronic disease patients within these 7 CEE countries.

## Methods

### Study design and setting

The PRICOV-19 study, set up by Ghent University (Belgium), investigated the impact of the pandemic on PC practices of 37 European countries and Israel. This multi-country study aimed to research the different dimensions of quality of care and how PC practices were organised during the COVID-19 pandemic to continue delivering high-quality care, and how task roles and well-being of healthcare providers changed in this period (13).

PRICOV-19 had a cross-sectional design and was administered electronically to staff working in PC practices. The questionnaire was intended for completion by one respondent in each practice, preferably a GP, or staff familiar with the practice organisation. The full list of practices was not available for the countries involved in this study, which hindered the planning of a specific recruitment strategy. The study aimed to sample 50+ practices per country, employing random or national convenience sampling. Data collection spanned from November 2020 (Romania and Hungary) to December 2021 (Ukraine). Collection duration varied from 2 months (Republic of Moldova) to 8 months (Ukraine), averaging 5 months across all countries (see Table 1).

The study protocol and data handling protocols are described in the Data Management Plan registered at Ghent University [22]. The questionnaire was developed at Ghent University in multiple phases, including a pilot study among 159 PC practices in Flanders (Belgium), and consisted of 53 items divided into six topics. More details are described in the study protocol [22]. The questionnaire was translated into seven languages of the countries included in this analysis following a standard procedure described in the protocol [22]. The Research Electronic Data Capture (REDCap) platform was used to host the questionnaire in all languages, send out invitations to the PC practices, and securely store the answers of the participants. We followed the STROBE guidelines for observational studies to ensure consistency and transparency in reporting the outcomes of this study.

### Data analysis

This paper focused on a subset of the questionnaire, including background information on characteristics of PC practices patient flow for COVID and non-COVID care, and communication with patients. Data checks were performed to assess and remove duplicate responses. Practices with less than 50% valid responses for an observation were excluded from the analyses. The Ghent University research team conducted initial data cleaning, followed by local teams in Chișinău at Nicolae Testemitanu State Medical University and at the Swiss Tropical and Public Health Institute performing data analysis.

We explored the binary outcome ‘PC practice having active patient follow up for patients with chronic disease’, which was defined as 1 for practices having contacted patients with a chronic condition who needed follow-up care since the beginning of the pandemic and as 0 for other practices.

Descriptive analyses involved counts and percentages for categorical variables and means with standard deviations (SD) were calculated for quantitative variables.

We built a mixed-effects logistic regression model to explore which factors were associated with active patient follow-up, accounting for clustering at country level. We calculated the adjusted odds ratios for having active patient follow-up when considering practice characteristics, the types of patient population and health system factors. Namely, we considered characteristics of the PC practices: rural vs. urban location of the practices, mean number of GPs, number of paid staff members including non-GPs, the use of video consultations and the availability of walk-in appointments during the pandemic, perceived time availability of GPs for reading guidelines since the beginning of the pandemic. We considered as characteristics of the patient population the presence of an above-average number of elderly patients and an above-average number of patients with chronic conditions within the practices and included an interaction term between these 2 variables. We also included health system factors in the model, such as perceived adequacy of government support offered to the practice during the pandemic and payment system. Categorical variables with more than two categories, were dichotomised or collapsed to limit the number of parameters in the regression models (see Table 1 in the appendix). We used backward selection of variables guided by the Aikaike Information Criterion (AIC) to derive the final model. Finally, we tested for heterogeneity of results across countries by comparing country-specific results using meta-analysis.

## Results

### Descriptive statistics

A total of 1893 PC practices in the seven countries submitted their responses to the PRICOV-19 study questionnaire. Four duplicate observations were dropped and 902 (47%) of observations were dropped due to a very high rate of incomplete responses having more than 50% of missing values within the variables of interest. A total of 987 PC practices within the seven countries were included in the analysis ranging between 67 in Moldova to 239 in Ukraine. Results are illustrated in Table 3. Moldova and Ukraine had a higher average number of patients registered per practice (or number of population covered) with a mean of 19611 (SD = 31791) patients in Moldova and 20891 (SD = 25246) patients per practice in Ukraine.

**Table 3.** Characteristics of PC practices in the 7 countries.

	Bulgaria	Czech Republic	Hungary	Moldova	Poland	Romania	Ukraine	Total
	*n* = 94	*n* = 105	*n* = 196	*n* = 67	*n* = 193	*n* = 91	*n* = 239	*n* = 973
**N. of GPs per PHC practice**	%	%	%	%	%	%	%	%
Solo practice (1 GP)	72.3	58.1	86.7	19.4	23.8	67.8	6.3	44.2
2–5 GPs	23.4	40.0	12.8	38.8	57.0	26.9	37.2	34.4
More than 5 GPs	3.2	1.9	0.0	40.3	19.2	3.2	52.7	20.1
Missing	1.1	0.0	0.5	1.5	0.0	2.1	3.8	1.42
**Location of facility**	%	%	%	%	%	%	%	%
Urban (city and suburbs)	73.4	72.4	75.0	40.3	67.9	87.1	69.9	70.72
Rural/small town/mixed	25.5	27.6	24.5	59.7	32.1	10.8	29.7	28.77
Missing	1.1	0.0	0.5	0.0	0.0	2.2	0.4	0.51
**Years of experience of respondent**	%	%	%	%	%	%	%	%
<10yrs	27.7	44.8	10.7	20.9	13.0	9.7	40.2	24.1
10–19yrs	30.9	21.9	23.0	22.4	11.9	26.9	22.2	21.6
20–29yrs	34.0	11.4	31.1	37.3	43.0	36.6	15.1	28.7
Above 29yrs	1.1	14.3	27.0	9.0	8.3	18.3	11.7	13.8
Missing	6.4	7.6	8.2	10.5	23.8	8.6	10.9	11.9
**Mean n. of staff (SD)**	1.5 (3.5)	1.7 (2.3)	1.2 (1.3)	13.6 (142.9)	3.5 (8.0)	1.5 (2.1)	10.5 (78.8)	24 (60.2)
**Mean n. of patients per practice (SD)**	2335 (1890)	2070 (701)	1872 (623)	19611 (31790)	6114 (4980)	2065 (790)	20891 (25246)	8401 (16944)
**Payment system of practice/GP**	%	%	%	%	%	%	%	%
Fee for service, other	10.6	10.5	12.2	22.4	3.6	49.5	36.0	20.2
Capitation	89.4	89.5	86.7	70.2	95.9	47.3	56.9	77.0
Missing	0.0	0.0	1.0	7.5	0.5	3.2	7.1	2.8
**Video consultations implemented since the pandemic**	%	%	%	%	%	%	%	%
Yes (less than once a week or more frequently)	58.5	76.2	63.3	47.8	70.0	63.4	68.6	51.9
No, Never	41.5	22.9	36.7	47.8	29.5	35.5	28.8	46.9
Missing	0.0	1.0	0.0	4.5	0.5	1.1	2.5	1.2
**Walk-in hours**	%	%	%	%	%	%	%	%
**Yes**	88.3	36.2	33.2	91.0	72.5	46.2	15.1	33.9
No	9.6	62.9	66.3	6.0	24.9	53.8	79.5	63.6
Missing	2.1	0.1	0.5	3.0	2.6		5.4	2.4
**Adequate support is provided by the government for the proper functioning of this practice.**	%	%	%	%	%	%	%	%
Yes	28.7	8.6	18.9	50.8	13.5	34.4	26.4	23.1
No	63.8	84.8	73.5	44.8	78.2	52.7	55.7	66.5
Missing	7.5	6.7	7.7	4.5	8.3	12.9	18.0	10.4
**Patient population: Elderly**	%	%	%	%	%	%	%	%
Above average	30.9	41.0	40.3	23.9	32.6	30.1	43.9	36.8
Average or below average	66.0	59.1	59.2	73.1	64.8	66.7	48.1	59.9
Missing	3.2	0.0	0.5	3.0	2.6	3.2	8.0	3.3
**Patient population with Chronic diseases**	%	%	%	%	%	%	%	%
Above average	38.3	29.5	31.1	31.3	33.2	47.3	59.4	40.4
Average or below average	61.7	68.6	64.8	65.7	63.7	50.5	33.5	55.8
Missing	0.0	1.9	4.1	3.0	3.1	2.2	7.1	3.8
**Active follow-up of patients with chronic conditions**	%	%	%	%	%	%	%	%
Yes	67.0	30.5	71.9	91.0	60.6	72.0	78.7	67.8
No	20.2	64.8	26.0	7.5	32.1	26.9	6.3	24.8
Missing	12.8	4.8	2.0	1.5	7.3	1.1	15.1	7.4

In 628 (63.6%) of responding PC practices walk-in hours were maintained during the pandemic with a great variation across countries, ranging from 24.8% in Poland to 91% in Moldova (see Table 2). Since the beginning of the pandemic, 46.9% of practices had implemented video consultations with the highest percentages seen in Ukraine (68.2%) and Romania (63.4%). The support given by the government was perceived to be adequate by 23.1% of respondents ranging from 8.6% in the Czech Republic to 34.4% in Romania.

**Table 2.** Dates of start and end of data collection in all countries and duration of data collection in months.

Country	Start data collection	End data collection	Months data collection
Bulgaria	15-Feb-21	31-Jul-21	5
Czech Republic	30-Nov-20	06-Mar-21	4
Hungary	25-Apr-21	13-Aug-21	4
Republic of Moldova	10-Feb-21	22-Apr-21	2
Poland	05-Feb-21	22-Aug-21	6
Romania	26-Nov-20	27-May-21	6
Ukraine	06-Apr-21	30-Dec-21	8

Overall 669 (67.8%) of practices responded that they actively followed up patients who needed chronic care. 399 practices (40.4%) responded that they have a higher than average number of patients with chronic diseases compared to other practices. Similar percentages were observed for having an above average number of elderly patients compared to the number of patients with chronic diseases.

Table 4 shows the adjusted odds ratios and AIC-values of the different logistic mixed effects models of active patient follow-up obtained in the backward selection process. In the final model (Model IV), there were positive associations between active patient follow up and (a) the mean number of doctors (aOR = 1.18 per additional doctor, p-value = 0.005), (b), having an above average elderly patient population (aOR = 1.36, p-value = 0.110), (c), having an above average patient population with chronic disease but a below average elderly population (aOR = 3.13, p-value = 0.006), (d) having the perception of being adequately supported by the government (aOR = 2.35, p-value = 0.001) and (e) the perception of having enough time for reading guidelines (aOR = 0.008, p-value = 1.71). Testing for heterogeneity of estimates across countries by comparing country-specific results by using meta-analysis, all p-values of the test of heterogeneity were larger than 0.5 for all variables of the final model (see Table 5).

**Table 4.** Results of backward selection of logistic mixed effects models for performing active patient follow up including random effects by country*.

Logistic Mixed Effect Models, Odds Ratio (OR) Fixed Effect Estimate (95% CI) for performing active patient follow up						
Independent variables	Model I: aOR Fixed Effect Estimate (95% CI)	Model II: aOR Fixed Effect Estimate (95% CI)	Model III: aOR Fixed Effect Estimate (95% CI)	Model IV: aOR Fixed Effect Estimate (95% CI)	Model V: aOR Fixed Effect Estimate (95% CI)	Model VI: aOR Fixed Effect Estimate (95% CI)
**Characteristics of the GP Practice**						
**Mean n. of GPs**	1.2 (1.06–1.36)*	1.2 (1.06–1.36)*	1.2 (1.06–1.36)*	1.2 (1.06–1.36)*	1.18 (1.05–1.32)*	1.18 (1.05–1.33)*
**Practice location**						
Urban	Ref.					
Rural	1.05 (0.69–1.58)					
**Number of staff in practice**						
Up to 5 staff	Ref.					
≥6 staff	0.77 (0.41–1.42)	0.76 (0.41–1.41)	0.76 (0.41–1.41)	0.76 (0.41–1.41)		
**Available Walk-in Consultations**						
Not available	Ref.					
Available	1.27 (0.85–1.91)	1.28 (0.85–1.91)	1.28 (1–1.91)	1.28 (0.85–1.91)	1.29 (0.86–1.93)	
**Available Video Consultations**						
Not implemented	Ref.					
Implemented	1.41 (0.97–2.06)	1.41 (0.97–2.07)	1.41 (1–2.06)	1.41 (0.97–2.06)	1.42 (0.98–2.08)	1.41 (0.97–2.05)
**Available time of the GP to review guidelines and literature**						
Not available	Ref.					
Available	1.71 (1.14–2.55)*	1.7 (1.14–2.54T)*	1.7 (1–2.54)*	1.7 (1.14–2.54)*	1.66 (1.12–2.47)*	1.71 (1.15–2.54)*
**Types of Patient Population**						
**Patient population Elderly**						
Below or approximately average	Ref.					
Above average	1.33 (0.67–2.64)	1.33 (0.67–2.65)	1.33 (1–1.94)	1.33 (0.91–1.94)	1.32 (0.91–1.93)	1.36 (0.93–1.98)
**Patient population: chronic disease**						
Below or approximately average	Ref.					
Above average	1 (0.49–2.07)	0.999 (0.48–2.06)				
**Patient population: Interaction term chronic disease** * **elderly population**						
Chronic diseases above average + Elderly population above average	Ref.					
Chronic diseases Above average + Elderly population below or average	3.07 (1.04–9.02)*	3.08 (1.05–9.06)*	3.08 (1.37 −6.90)*	3.08 (1.37–6.9)*	3.1 (1–6.96)*	3.13 (1.39–7.01)*
**Health System Factors**						
**Perceived adequacy of government support**						
Not adequate	Ref.					
Adequate	2.29 (1.4–3.76)*	2.3 (1.41–3.77)*	2.3 (1.41–3.77)*	2.3 (1.41–3.76)*	2.3 (1.41–3.75)*	2.35 (1.44–3.83)*
**Payment System**						
Fee-for-service, or other type of payment	Ref.					
Capitation	0.99 (0.58–1.7)	1 (0.58–1.71)	1 (0.58–1.71)			
AIC	776.0516	774.0981	772.0981	770.0982	768.8453	768.3842

** Backward selection was based on the Akaike information criterion (AIC).

* Significant at 5% level.

**Table 5.** Meta-analysis of the country-specific effect estimates obtained by geographjc stratification of the final model of active patient follow-up. (see Table 1 for complete list of variables).

Independent variable	**I-squared** *	**P-value** **
Above average patients with chronic disease and below average n. of elderly patients	0.00%	0.74
Above average elderly patients	0.00%	0.76
Perceived adequate government support	0.02%	0.52
Time to read guidelines	0.00%	0.48
Availability of video consultations	0.00%	0.73

* Here, I-squared is an estimate of the proportion of variance in country-specific estimates which is unexplained by chance.

** Chi-squared test of heterogeneity.

## Discussion

### Main findings

We found a significant association between active patient follow-up and specific service delivery and health system factors. Practices with GPs that have enough time to read guidelines were positively associated with active patient follow-up across countries. This corroborates findings from another systematic review looking at different determinants of follow-up care for cancer, which found that GPs were more likely to follow-up with patients after receiving appropriate training and guidelines in support of case management [23]. Having more GPs in a practice has also been shown to be positively associated with patient follow-up also in other studies due to having less time pressure and a more manageable workload that allow for active follow-up [13, 24]. The availability of video consultations or walk-in hours did not show a significant association with having patient follow-up. A review of reviews of remote consultations found that they were effective in monitoring, and for implementing psychological and health behaviour change interventions and for assessing some chronic conditions related to cardiovascular and respiratory diseases but evidence was weak for other types of conditions [25]. When assessing the characteristics of the patient population and whether better follow-up was associated with a higher than average number of elderly or chronic patients, we found that the combination of having a higher than average number of chronic patients but a below average number of elderly patients, was associated with better follow-up. This could be because GPs might prioritise follow-up of younger patients with chronic diseases, or because younger patients with chronic diseases, or their families, might be more likely to request follow-up. From a health system perspective, a perceived adequate government support was associated with active follow-up. Government support may also be linked to the rapid uptake of new technologies as evidenced in other studies across Europe, which showed that a centralised and rapid deployment of remote consultation software, enabled the use of new technologies in PC practices [26].

An increase in medical services provided and improvement of patient outcomes have been linked to GP incentive (fee-for-service) [27] though in our study, we did not observe any association between patient follow-up with the type of payment system in place in practices (fee-for-service or capitation).

A PRICOV series paper based on the overall database evaluated a composite outcome from four variables concerning patient follow-up (outlined as outreach work) across 38 countries. The variables encompassed active follow-up for psychologically vulnerable patients, those with chronic conditions, individuals experiencing domestic violence, and the use of EMR systems for at least one of these patient groups [28]. In this study, 62% of practices actively followed-up patients with chronic conditions, slightly lower than in our sample. This study showed that a significant association was found between performing outreach work with having an administrative assistant, while other variables, such as number of staff, patient composition and payment system were not significantly associated with the outcome. Comparability with our study is limited though, due to the difference in the outcome definition, as we focused exclusively on active follow-up of patients with chronic conditions and different determinants of follow-up.

### Strengths and limitations

To our knowledge, this is the first study comparing active patient follow-up in the CEE region, which have undergone important efforts in improving the PHC system and universal health coverage for their populations. The survey was designed and validated through rigorous methods and was the largest survey conducted among PC practices to assess the impact of COVID on several dimensions pertaining to service delivery, with data collection methods tested in 38 countries. The study highlights the importance of contextual factors when planning for health services in crises, as differences were observed across countries which may be difficult to explain through quantitative data. The study’s cross-sectional design limits causal inference. Additionally, responder bias and convenience sampling may affect generalisability of the results. Despite these constraints, the study offers valuable insights into primary care practices during the COVID-19 pandemic in seven CEE countries, illustrating factors influencing active patient follow-up.

### Implications

In times of crisis, PC practices should be adequately supported from the government to re-organize their practices to better follow up patients. Policy-makers should also implement solutions to adequately staff PC practices to decrease their workload, to better deliver services according to the practice needs.

## Conclusion

In the 7 CEE countries, nearly 70% of practices reported actively following-up with patients having chronic disease and, active patient follow up was significantly associated with factors related to the health system and specific factors related to the PC practice such as number of staff, time availability of staff, perceived adequacy of government support and composition of the patient population with chronic diseases. Implementation of measures such as video consultations and the type of payment services showed no association, likely due to contextual factors which need to be further explored.

## Appendix

**Appendix Table 1.** Description of data modifications applied to single variables.

Variable	Data modifications
Urban vs. Rural location of the PC practice	Dichotomization of original variable which had 5 levels: with PC practices located in big inner city, suburbs and small towns as ‘urban’ and practices located in rural or mixed-rural urban areas, as ‘rural’
Time availability of GPs for reading guidelines since the beginning of the pandemic	The question was formulated as ‘In this practice, there is enough protected time provided in the agenda(s) of GPs for reviewing guidelines or going through relevant and reliable scientific literature’ and the responses were collected on a five point scale from strongly disagree to strongly agree. Responses were coded as 0 if the respondent answered ‘Disagree/Strongly disagree/Neutral’ and as 1 if the respondent answered ‘Agree/Strongly Agree’
Perceived adequacy of government support offered to the practice during the pandemic	The question was formulated as: ‘Adequate support is provided by the government for the proper functioning of this practice’. Responses were originally coded on a Likert scale Responses were coded as 0 if the respondent answered ‘Disagree/Strongly disagree/Neutral’ and as 1 if the respondent answered ‘Agree/Strongly Agree’
Type of payment system of the PC practice	The original variable contained three levels: capitation, fee-for-service and other. Responses were dichotomised to capitation =1 and fee-for-service and other =0
